# Transient enalapril attenuates the reduction in glomerular filtration rate in prenatally programmed rats

**DOI:** 10.14814/phy2.13266

**Published:** 2017-04-24

**Authors:** Asifhusen Mansuri, Ayah Elmaghrabi, Issa Alhamoud, Susan K. Legan, Jyothsna Gattineni, Michel Baum

**Affiliations:** ^1^ Department of PediatricsUniversity of Texas Southwestern Medical Center at DallasDallasTexas; ^2^ Internal MedicineUniversity of Texas Southwestern Medical Center at DallasDallasTexas

**Keywords:** Glomerular filtration rate, hypertension, postnatal programming, prenatal programming

## Abstract

A maternal low‐protein diet has been shown to program hypertension and a reduction in glomerular filtration rate in adult offspring. This study examined the effect of continuous administration of enalapril in the drinking water and transient administration of enalapril administered from 21 to 42 days of age on blood pressure and glomerular filtration rate (GFR) in male rats whose mothers were fed a 20% protein diet (control) or a 6% protein diet (programmed) during the last half of pregnancy. After birth all rats were fed a 20% protein diet. Programmed rats (maternal 6% protein diet) were hypertensive at 15 months of age compared to control rats and both continuous and transient administration of enalapril had no effect on blood pressure on control offspring, but normalized the blood pressure of programmed offspring. GFR was 3.2 ± 0.1 mL/min in the control group and 1.7 ± 0.1 mL/min in the programmed rats at 17 months of age (*P* < 0.001). The GFR was 3.0 ± 0.1 mL/min in the control and 2.7 ± 0.1 mL/min in the programmed group that received continuous enalapril in their drinking water showing that enalapril can prevent the decrease in GFR in programmed rats. Transient administration of enalapril had no effect on GFR in the control group (3.2 ± 0.1 mL/min) and prevented the decrease in GFR in the programmed group (2.9 ± 0.1 mL/min). In conclusion, transient exposure to enalapril for 3 weeks after weaning can prevent the hypertension and decrease in GFR in prenatal programmed rats.

## Introduction

There is increasing evidence that small for gestational age neonates and neonates that are born very prematurely are at risk for chronic kidney disease in later life (White et al. 2009). Small for gestational age neonates are born with a reduction in nephron number compared to those of normal birth weight (Hinchliffe et al. 1992; Manalich et al. 2000; Hughson et al. 2003). A study examining glomerular number in infants who died in the first 2 weeks of age and who were over 36 weeks gestation, a time when nephrogenesis is complete, showed that there was a direct correlation between birth weight and glomerular number (Manalich et al. 2000). Examining autopsy specimens Hughson et al. (2003) found that there was an approximately 250,000 increase in the number of glomeruli for each kilogram increase in birth weight. In addition, very premature neonates have nephron development for only 40 days after birth, which may terminate nephrogenesis prematurely and result in neonates with a diminished nephron endowment (Rodriguez et al. 2004). This is likely due to the fact that the extrauterine postnatal environment of a very premature infant does not provide the same nutrition and nurturing milieu as that in the mother's womb.

According to the Brenner hypothesis, a reduced nephron endowment is a risk factor for increased glomerular capillary pressure, proteinuria, and chronic kidney disease (Brenner and Chertow 1993, 1994). Indeed, there is evidence that low birth weight and very premature neonates are at risk for progressive renal disease. Australian Aboriginal children born less than 2.5 kg have lower kidney volumes, an indirect marker for glomerular endowment, than those born with a normal birth weight (Spencer et al. 2001). When assessed as young adults, low birth weight Aborigines have an almost threefold likelihood of an increased albumin/creatinine ratio, a harbinger of chronic kidney disease (Hoy et al. 1999). Low birth weight has been shown to result in a decrease in estimated glomerular filtration rate even when assessed in school‐aged children (Lopez‐Bermejo et al. 2008). A birthweight of less than 2.5 kg is a risk factor for developing end stage renal disease in whites and African Americans compared to those weighing 3–3.5 kg (odds ratio 1.4) (Lackland et al. 2000). A Norwegian study demonstrated that those individuals born at less than the 10th percentile for weight have a 1.7‐fold relative risk of developing end stage renal disease compared to those born between the 10th and 90th percentile (Vikse et al. 2008). There is also evidence that very premature infants are at risk for developing proteinuria and chronic kidney disease due to focal and segmental glomerulosclerosis (Keijzer‐Veen et al. 2005; Hodgin et al. 2009).

Animal studies have been designed to mimic some of the insults that result in small for gestational age neonates including maternal caloric or protein deprivation, uteroplacental insufficiency, and prenatal exposure to glucocorticoids. Most studies have found that these prenatal insults result in an ~30% reduction in glomerular number with a concordant increase in blood pressure in programmed rats (Celsi et al. 1998; Ortiz et al. 2001; Vehaskari et al. 2001; Wlodek et al. 2008; Habib et al. 2011a). Studies looking at glomerular filtration rate in 1.5‐year‐old offspring of rats whose mothers were fed a low‐protein diet during the last half of pregnancy or a 50% intrauterine food restriction throughout pregnancy have a decrease in glomerular filtration rate compared to control rats (Lucas et al. 2001; Lozano et al. 2015). Interestingly, there does not appear to be a reduction in glomerular filtration rate in rats with comparable insults studied in the first 6 months of age indicating that the reduction in glomerular filtration rate is not simply a reflection of the reduction in nephron number (Martins et al. 2003; Ortiz et al. 2003; Siddique et al. 2014; Lozano et al. 2015). Several studies have shown that transient administration of enalapril to programmed rats at about the time of weaning can have a sustained effect on blood pressure for several weeks after the enalapril has been discontinued (Langley‐Evans and Jackson 1995; Sherman and Langley‐Evans 1998, 2000; Mansuri et al. 2015). We have recently demonstrated that transient administration of enalapril can reprogram the intrarenal renin‐angiotensin system and have a sustained effect on blood pressure in programmed rats for at least 20 weeks after discontinuing the enalapril when administered between 3 and 6 weeks of age (Mansuri et al. 2015). It is our hypothesis that early administration of enalapril will have a lasting effect by reprogramming the rats whose mothers were fed a low‐protein diet to prevent the decrease in glomerular filtration rate (Lozano et al. 2015). The present study was undertaken to examine if treatment of offspring with enalapril, an angiotensin converting enzyme inhibitor, either from weaning at 21 days of age until study at 1.5 years or transient administration of enalapril from days 21 to 42 days of age would have an impact on the decrease in glomerular filtration rate and hypertension in programmed rats.

## Methods

### Animals

Pregnant Sprague Dawley were fed a 20% protein diet until day 12 of gestation. At day 12 they were either fed an isocaloric 6% protein diet or they remained on the 20% protein diet until they delivered. The diets had the same mineral and vitamin content. Immediately after delivery all rats were fed a 20% protein diet as were the offspring after weaning. This protocol has been used by our laboratory and others previously (Vehaskari et al. 2001; Habib et al. 2011b; Mansuri et al. 2015). In an previous study we showed that this protocol did not affect the size of the litters (11.4 ± 0.6 pups in the 20% vs. 13.0 ± 0.5 pups in the 6% group, *P* = ns) (Habib et al. 2011b). After weaning the rats were divided into three groups and only two rats per group were used from any litter. Some of the rats were administered enalapril starting at 21 days of life. Nephrogenesis in the rat is complete by approximately 10 days of age and thus enalapril did not affect nephrogenesis (Kavlock and Gray 1982; Tufromcreddie et al. 1995). Males were studied to reduce variability (Alexander 2003; Ortiz et al. 2003; Woods et al. 2005; Moritz et al. 2009). These studies were approved by the IACUC of the University of Texas Southwestern Medical Center and the animals were cared for according to the Guide for the Care and Use of Laboratory Animals (NRC).

At 21 days of age the rats were weaned and divided into six groups:

1. 20% Vehicle (20%) – The mother was fed a 20% protein diet throughout pregnancy. The weaned rat was given water that contained 4 cc/L of ethanol which was the vehicle used to dissolve the enalapril. Vehicle was in the drinking water until the time of study.
2. 6% Vehicle (6%) – The 6% group was comprised of rats whose mothers were fed a 6% protein diet from day 12 of pregnancy until they delivered. The diet was subsequently changed to a 20% protein diet. After weaning the rats were given water that contained 4 cc/L of ethanol until the time of study.
3. 20% Continuous Enalapril (20% CE) – After weaning the 20% protein fed rats were administered enalapril (100 mg/L) in their drinking water continuously until the time of study. Enalapril (100 mg) was dissolved in 4 mL of ethanol. This protocol has been used by others from weaning until 16 weeks of age (Manning and Vehaskari 2005).
4. 6% Continuous Enalapril (6% CE) – After weaning the 6% protein group was treated with enalapril (100 mg/L) in their drinking water continuously until the time of study.
5. 20% Transient Enalapril (20% TE) – After weaning the 20% rats were given water that contained enalapril (100 mg/L) in their drinking water for 21 days. After 21 days the enalapril was discontinued and the rats continued on the ethanol vehicle in their drinking water. We have used this protocol previously in rats up to 6 months of age (Mansuri et al. 2015).
6. 6% Transient Enalapril (6% TE) – After weaning the 6% rats were administered enalapril (100 mg/L) in their drinking water for 21 days. After 21 days the enalapril was discontinued and the rats continued on the ethanol vehicle in their drinking water.

### Measurement of blood pressure

Blood pressure was measured at 15 months of age. The rats were trained for 4 days prior to the actual measurement of blood pressure by placing them in a Lucite tube and inflating the blood pressure cuff as would be performed during the actual measurement of blood pressure. The investigator measuring the blood pressure of the rats was blinded and did not know from which group the rat originated (Mizuno et al. 2013, 2014; Mansuri et al. 2015). Blood pressures were measured using the CODA Blood Pressure Non‐Invasive Pressure Analyzer (Kent Scientific Corporation, Torrington, CT). The instrument uses a volume pressure recording which correlates well with measurements made using telemetry (Feng et al. 2008). The mean of at least 6 recordings was used as the blood pressure for the rat.

### Glomerular filtration rate

Glomerular filtration rate was measured at 17 months of age using the same inulin clearance protocol previously used in our laboratory (Ortiz et al. 2001, 2003; Lozano et al. 2015). We measured glomerular filtration rate in 17‐month‐old rats as we have previously found that programmed rats survive until this age but have a significant decrease in glomerular filtration rate (Lozano et al. 2015). Rats were anesthetized using 100 mg/kg of Inactin which was injected into the peritoneal cavity. The neck, groin, and chest were shaved with an electric shaver and the rats were then placed on a servo‐controlled table to maintain a body temperature of 37°C. Polyethylene tubing was used as catheters that were placed in the carotid artery and femoral vein. A tracheostomy was then performed and polyethylene tubing was placed into the trachea. The bladder was exposed with a midline 2 cm incision 2–3 cm above from the pubic bone. An 18‐gauge needle (Becton, Dickinson and Company, Franklin Lakes, NJ) was used to puncture the bladder on the ventral side of the bladder neck. Polyethylene tubing was then introduced into the bladder through the puncture site. The site was checked for leakage and catheter was rinsed with 0.9% sterile saline solution containing 10 units of heparin/mL. The catheter was then secured to allow free flow of urine that could be collected and quantitated.

^3^H‐methoxy inulin was exhaustively dialyzed and then dried. Six *μ*Ci of ^3^H‐methoxy inulin was administered intravenously as a bolus in normal saline. Inulin was then administered at a constant rate of 16 *μ*Ci per h at 0.6 mL/h/100 gm of body weight. After 1 h of equilibration, five 30‐min urine samples were collected. Blood samples were collected at the midpoint of the urine collection for the measurement of inulin. Blood and urine samples were centrifuged and 50 *μ*L of serum and 50 *μ*L of urine were taken for measurement of ^3^H‐inulin using liquid scintillation counter (Tri‐Carb 2100TR Liquid Scintillation Analyzer, Perkin‐Elmer Life Sciences Waltham MA). The mean glomerular filtration rate from five collections was used as the glomerular filtration rate of that rat.

After measurement of glomerular filtration rate, two cc's of blood was removed for hormone assays from the arterial line, and the rat was sacrificed. The heart and kidneys were removed, blotted dry of blood and weighed. One kidney was placed in 10% formalin for histology. A portion of one kidney was stored at −80°C for measurement of angiotensin II and collagen abundance.

### Kidney angiotensin II

Kidney angiotensin II was assayed in a similar fashion as we have previously described (Dagan et al. 2010). Briefly, kidney slices were weighed and then ground in cold methanol. The homogenates were centrifuged at 4°C for 10 min at 1000 *g*. The supernatants were transferred to new tubes and the methanol was evaporated to dryness using a stream of nitrogen. For extraction of angiotensin II, each sample was resuspended in water and loaded on individual, conditioned and equilibrated Strata Phenyl cartridges (Phenomenex, Torrance, CA). After washing with water, the angiotensin II was eluted with methanol and evaporated under a stream of nitrogen. The angiotensin II was reconstituted in EIA buffer and measured using an Angiotensin II Enzyme Immunoassay Kit from SPI‐Bio (Montigny le Bretonneux, France) in accordance with manufacturer's instructions. The total angiotensin II was normalized per gram of tissue.

### Blood assays

The angiotensin II was determined using the Angiotensin II Enzyme Immunoassay Kit from SPI‐Bio (Montigny le Bretonneux, France) in accordance with manufacturer's instructions. Renin activity was measured by an ELISA assay using a Renin Activity ELISA (ALPCO, Salem, NH) and aldosterone was measured per manufacturer's instructions by an Aldosterone EIA Kit (Enzo, Plymouth Meeting, PA).

### Collagen abundance

Renal collagen content was measured in the 17‐month‐old rats after measurement of glomerular filtration rate by measuring hydroxyproline using a Hydroxyproline Colorimetric Assay Kit (BioVision Incorporated, Milpitas, CA). Approximately 10 mg of frozen kidney cortex was weighed and assayed for hydroxyproline per manufacturer's instructions. Briefly, tissue was homogenized, digested with 6N hydrochloric acid and heated at 120°C for 3 h. Samples were evaporated to dryness and hydroxyproline content was assayed per instructions. Collagen abundance was extrapolated from the hydroxyproline content assuming that collagen contained 12.7% hydroxyproline by weight. Results were expressed as *μ*g collagen/mg kidney.

### Histologic assessment of interstitial fibrosis and glomerular injury

The kidney was cut in 5 *μ*m slices. Glomerular injury was assessed with slides stained with periodic acid‐Schiff at 300× magnification. We used the same scale to assess glomerulosclerosis and mesangial expansion as described by Raij et al. (1984). Mesangial matrix expansion was graded from 0 to 4 for each of 20 random glomeruli per slide based on the amount of periodic acid‐Schiff staining. Glomerulosclerosis was graded from 0 to 4 based on the amount of glomerular involvement in 20 random glomeruli per slide where sclerosis of 25% of the glomerulus was scored 1 and 50% scored 2 etc. The average score for each slide for both glomerulosclerosis and mesangial matrix expansion was multiplied by 100 to give a score from 0 to 400. Slides were examined by two different investigators in a blinded fashion and the mean of their score was used as the mean for that slide.

Interstitial fibrosis was assessed using kidney slices stained with picrosirius red with and without polarization in the total field (minus large blood vessels and glomeruli) at 250× magnification (Oda et al. 2001) An Axioplan‐2 Zeiss microscope with a Zeiss Axiocam MRC3 camera (Carl Zeiss Thornwood, NY) was used to photograph the slides without polarization. Interstitial collagen was also measured under polarized light to determine collagen I and III abundance (Junqueira et al. 1978, 1979; Oda et al. 2001; Farris et al. 2011) with a Nikon Eclipse TE 2000‐U microscope and a DS‐U3 digital camera (Nikon Instruments, Japan). Ten images of cortex and 10 images of outer medulla were analyzed using NIS‐Elements BR 3.2 software to quantify fibrosis (Chen et al. 2011). The percent of the image analyzed that was stained with picrosirius red and picrosirius red with polarized light was compared between the groups.

### Chemicals

All chemicals were purchased from Sigma Chemical Company (St. Louis, MO) unless otherwise designated.

### Statistical analysis

Data are reported as mean ± standard error of the mean. Comparisons between the groups were assessed using analysis of variance with a post hoc Bonferroni test. A *P* < 0.05 was considered significant.

## Results

### Effect of a prenatal low‐protein diet and enalapril on blood pressure

**Figure 1:**
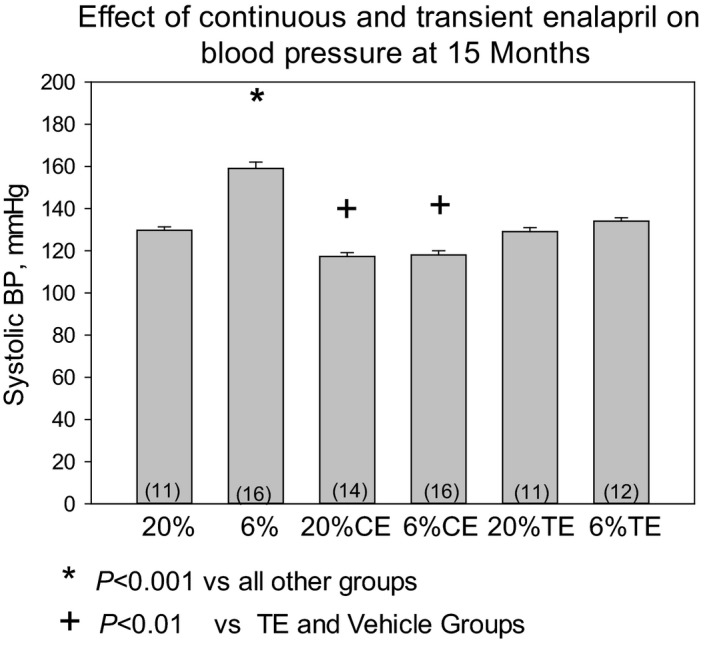
Effect of prenatal protein diet and postnatal enalapril on blood pressure at 15 months of age: Systolic blood pressure was measured at 15 months of age by tail cuff in rats whose mothers were fed a 20% protein diet (20%) or a 6% protein diet (6%) during the last half of pregnancy. All mothers were fed a 20% protein diet after birth as were the offspring after weaning. The rats had either vehicle, enalapril (100 mg/L) from 21 days of age until the time of study (CE), or had enalapril (100 mg/L) from day 21 to day 42 days of life (21 days total) (TE). The transient enalapril group was then administered vehicle until time of study. Blood pressure was measured in a blinded fashion using tail cuff in trained rats. Data are reported as mean ± standard error of the mean and analyzed using analysis of variance with a post hoc Bonferroni test. *P* < 0.05 is considered statistically significant. The number of rats is shown in parenthesis.

In the first series of experiments we examined if maternal dietary protein deprivation during the last half of pregnancy would have a lasting effect on blood pressure and if continuous or transient enalapril would affect blood pressure in control or the programmed group. As shown in Figure 1, the 6% vehicle group had a significantly higher blood pressure than the 20% vehicle group at 15 months of age. Continuous administration of enalapril to the 6% and 20% groups resulted in lower blood pressures compared to all other groups. Interestingly, transient administration of enalapril from days 21 to 42 of life normalized the blood pressure of the 6% group (6% TE) to a level comparable to the 20% vehicle group. In comparing all groups, the 6% vehicle group had a higher blood pressure than the other five groups (*P* < 0.001).

### Effect of prenatal programming and postnatal enalapril on body and kidney weight

The effect of prenatal programming and enalapril on body and kidney weight is shown in Table 1. As we had found previously, the 20% vehicle group weighed more than the 6% group at 17 months (Lozano et al. 2015). Both transient and continuous administration of enalapril also had an effect body weight and the 20% vehicle group weighed more than the 6% and 20% continuous and transient enalapril groups.

**Table 1:** Effect of continuous and transient enalapril on body and kidney weight 17 months of age

	Body wt (gm) 17 months	Kidney Wt (gm)	Kidney(gm)/100 gm BW
20%	631 ± 14^a^	1.36 ± 0.03	0.22 ± 0.01^c^
6%	532 ± 8	1.27 ± 0.05	0.24 ± 0.01^d^
20% CE	519 ± 14	1.38 ± 0.04	0.27 ± 0.01
6% CE	484 ± 7	1.35 ± 0.03	0.28 ± 0.01
20% TE	562 ± 22^b^	1.39 ± 0.03	0.25 ± 0.01
6% TE	521 ± 13	1.36 ± 0.04	0.27 ± 0.01

*n* = 8 in each group.

a *P* < 0.05 versus all groups.

b *P* < 0.005 versus 6% CE.

c *P* < 0.05 versus 6% CE, 20% CE, 6% TE.

d *P* < 0.05 versus 6% CE.

The kidney weight was comparable between all of the groups. However, the 20% vehicle kidney/body weight was less than the 6% continuous and 6% transient enalapril group and the 20% continuous enalapril group. The 6% vehicle group had a lower kidney weight/body weight than the 6% continuous enalapril group.

### Effect of prenatal programming and postnatal enalapril on GFR

**Figure 2:**
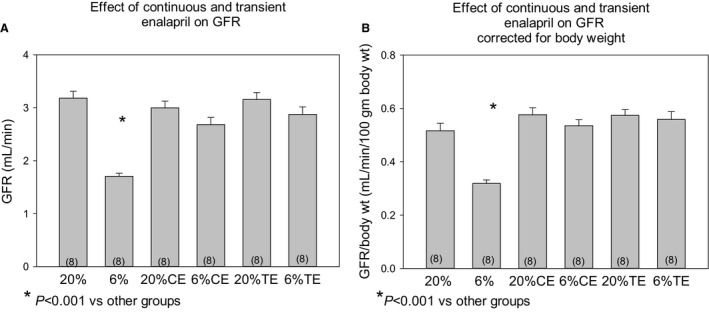
Effect of prenatal protein diet and postnatal enalapril on glomerular filtration rate at 17 months of age. The glomerular filtration rate (mL/min) was assessed using inulin clearance in 17‐month‐old offspring of mothers who were fed either a 6% or a 20% protein diet during the last half of pregnancy after which all rats were fed a 20% protein diet. Glomerular filtration rate was measured using inulin clearance at 17 months of age. Rats were either given vehicle (20%) and (6%), continuous enalapril (CE) in their drinking water (100 mg/L) or enalapril from days 21 to 42 of life followed by vehicle (TE). Glomerular filtration rate is shown in 2A in mL/min and 2B where glomerular filtration rate was corrected for body weight. Data are reported as mean ± standard error of the mean and analyzed using analysis of variance with a post hoc Bonferroni test. *P* < 0.05 is considered statistically significant. The number of rats is shown in parenthesis.

Glomerular filtration rate (GFR) was measured using inulin clearance at 17 months of age. The 6% group whose mothers were fed a low‐protein diet during the last half of pregnancy had a lower GFR than the offspring of rats who were fed a 20% protein diet throughout pregnancy. As shown in Figure 2A, the glomerular filtration rate of the 6% group that received either continuous enalapril or transient enalapril from days 21 to 42 had a comparable GFR to the 20%, 20% CE and 20% TE groups. The GFR of the 6% group was lower than all of the other groups (*P* < 0.001). In Figure 2B, the GFR was normalized per 100 g body weight. The GFR/100 g body weight in the 6% group was lower than the other five groups. Thus, transient administration of enalapril can prevent the decrease in GFR in programmed rats.

### Effect of prenatal programming and enalapril on renal angiotensin II content and serum renin, angiotensin ii, and aldosterone levels

The serum renin, angiotensin II, and aldosterone levels are shown in Table 2. The renin activity was higher with continuous administration of enalapril but only reached statistical significance in the 20% continuous enalapril compared to the 20% vehicle and 6% vehicle. Serum angiotensin II was comparable in all the groups. Aldosterone was higher in the 6% vehicle group than in all of the other groups.

**Table 2:** Effect of continuous and transient enalapril on serum levels of renin, angiotensin II and aldosterone 17 months of age

	Serum renin activity (ng/mL/h)	Angiotensin II (pg/mL)	Aldosterone (pg/mL)
20%	21.2 ± 5.1	51.7 ± 17.6	664.2 ± 151.4
6%	38.9 ± 15.2	129.6 ± 37.0	1941.2 ± 121.2^b^
20% CE	227.3 ± 78.0^a^	259.4 ± 75.6	727.5 ± 110.6
6% CE	175.8 ± 50.3	260.7 ± 87.8	769.9 ± 252.7
20% TE	89.4 ± 47.6	96.3 ± 41.4	758.4 ± 202.6
6% TE	55.7 ± 12.4	58.8 ± 19.9	784.5 ± 197.5

a *P* < 0.05 versus 20% and 6%.

b *P* < 0.01 versus all other groups.

**Figure 3:**
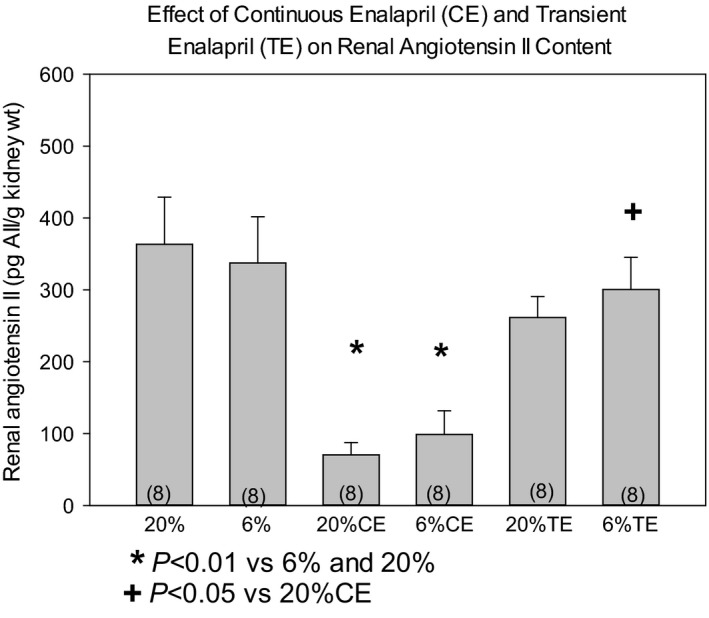
Effect of prenatal protein diet and postnatal enalapril on renal angiotensin II content at 17 months of age. After measurement of glomerular filtration rate the kidneys were immediately weighed and frozen until assay for Angiotensin II content. Data are reported as mean ± standard error of the mean and analyzed using analysis of variance with a post hoc Bonferroni test. *P* < 0.05 is considered statistically significant. The number of rats is shown in parenthesis.

To determine if the intrarenal renin angiotensin system was affected by continuous or transient enalapril, angiotensin II content (pg/g tissue weight) was measured. As shown in Figure 3, continuous enalapril lowered the renal angiotensin II content comparably in both the 6% and 20% to a level lower than the 20% and 6% vehicle groups. Programmed (6%) rats had comparable renal angiotensin II contents to the 20% rats. The 6% transient enalapril renal angiotensin II content was higher than the 20% continuous enalapril group. Transient administration of enalapril had no effect on the angiotensin II content of the kidneys compared to the vehicle‐treated rats.

### Effect of prenatal programming and enalapril on interstitial fibrosis and glomerular injury

The results of histologic analysis of interstitial fibrosis, glomerular injury and renal collagen content is shown in Table 3. Interstitial fibrosis was assessed using polarized and unpolarized light in the cortex and outer medulla. As we have previously found, there is relatively little interstitial fibrosis and no difference between the 17‐month‐old offspring of mothers fed a 6% protein diet and 20% protein diet (Lozano et al. 2015). The 20% group that was administered continuous enalapril had higher cortical interstitial fibrosis measured using polarized light than the 20% vehicle group. The cause for this difference is not clear. Mesangial expansion was comparable in all of the groups. The 6% vehicle group had a higher glomerulosclerosis score than the 20% vehicle group when assessed using an unpaired Student's *t* test consistent with glomerular injury in the programmed rats at 17 months of age. However, when comparing all of the groups together, the degree of glomerulosclerosis was mild and not different between the groups. The collagen content per milligram of tissue was also comparable between the groups. Thus, as we have previously found (Lozano et al. 2015), despite the marked decrease in GFR in the 6% vehicle group, there was little difference in interstitial fibrosis and collagen content.

**Table 3:** Effect of continuous enalapril and transient enalapril on renal collagen content, interstitial fibrosis, mesangial matrix expansion and glomerulosclerosis

	*μ*g collagen/mg tissue	Interstitial PS red (cortex) (%)	Interstitial PS red (outer medulla) (%)	Interstitial PS red polarized (cortex) (%)	Interstitial PS red polarized (outer medulla) (%)	Glomerular mesangial matrix expansion	Glomerulosclerosis
20%	6.8 ± 0.3	6.8 ± 0.5	5.1 ± 0.4	2.2 ± 0.1	2.0 ± 0.1	213.1 ± 28.1	31.3 ± 8.8
6%	6.7 ± 0.3	7.9 ± 0.6	6.4 ± 0.5	2.4 ± 0.2	2.7 ± 0.3	219.4 ± 30.1	65.3 ± 9.2^b^
20% CE	6.2 ± 0.3	7.1 ± 0.5	5.9 ± 0.4	3.3 ± 0.3^a^	2.8 ± 0.3	184.1 ± 39.4	43.4 ± 9.1
6% CE	5.4 ± 0.4	7.3 ± 0.5	5.8 ± 0.4	2.8 ± 0.2	2.5 ± 0.2	224.1 ± 23.5	40.3 ± 5.2
20% TE	6.4 ± 0.3	6.8 ± 0.4	5.6 ± 0.4	2.5 ± 0.2	2.1 ± 0.2	164.1 ± 27.3	38.4 ± 7.1
6% TE	5.8 ± 0.4	8.0 ± 0.7	6.0 ± 0.8	2.6 ± 0.3	2.4 ± 0.3	191.8 ± 30.3	43.8 ± 11.4

*N* = 8 in each group.

a *P* ≤ 0.05 versus 20% V.

b *P* < 0.05 versus 20% vehicle by unpaired Students *t* test.

## Discussion

This study examined whether continuous or transient administration of enalapril for 3 weeks after the time of weaning would affect blood pressure and GFR in programmed rats. We find that both continuous and transient administration of enalapril prevented the hypertension seen in offspring of rats whose mothers were fed a low‐protein diet during the last half of pregnancy. Interestingly, continuous and even transient administration of enalapril was able to prevent the decrease in glomerular filtration rate in programmed rats at 17 months of age.

Previous studies have shown that transient administration of enalapril or losartan, an angiotensin II receptor blocker, administered either between 2 and 4 weeks of age or 3 and 6 weeks of age resulted in a sustained decrease in blood pressure in prenatally programmed rats to levels comparable to controls for about 2 months after the drug was discontinued (Sherman and Langley‐Evans 1998, 2000; Manning and Vehaskari 2005). We have recently shown that the effect of transient administration of enalapril from 3 to 6 weeks of age resulted in normalization in blood pressure in 6% rats to levels comparable to 20% rats when the rats were studied at 6 months of age (Mansuri et al. 2015). The present study showed that the transient effect of enalapril on blood pressure in prenatal 6% programmed rats can be sustained for over a year after discontinuation of the drug. This is consistent with reprogramming of the factor or factors that mediate hypertension in programmed rats.

The effect of prenatal programming on the systemic and intrarenal renin‐angiotensin system in adult offspring has been examined previously (Vehaskari and Woods 2005; Kett and Denton 2011). Focusing on studies where mothers were fed a low‐protein diet during pregnancy, plasma renin activity was found to be lower in programmed than control at 4 and 8 weeks of age (Vehaskari et al. 2001), but higher in programmed rats at 16 weeks and 11 months of age (Manning and Vehaskari 2001, 2005), while others found no difference between control and programmed rats at 13 weeks (Langley‐Evans and Jackson 1995) or 6 months of age (Mansuri et al. 2015). Plasma angiotensin II levels are comparable in offspring of rats whose mothers were fed a low‐protein diet at 4 weeks, 13 weeks and at 6 months of age (Langley‐Evans and Jackson 1995; Vehaskari et al. 2004; Mansuri et al. 2015). Aldosterone is higher in programmed rats than controls at 1, 2, and 4 months but not at 6 months (Vehaskari et al. 2001; Cheng et al. 2012; Mansuri et al. 2015). In the present study at 17 months of age, we found higher serum renin levels in the continuous enalapril groups than the transient enalapril groups and the vehicle groups, however only the 20% continuous enalapril group was statistically greater than the 6% and 20% vehicle groups. This was comparable to our findings examining these groups at 6 months of age (Mansuri et al. 2015). As we have previously found at 6 months, there was no difference in angiotensin II levels between these groups (Mansuri et al. 2015). While we did not see an increase in aldosterone levels comparing these groups at 6 months of age (Mansuri et al. 2015), the aldosterone levels were higher in the 6% group than all other groups in this study at 17 months of age. Thus, both continuous and transient enalapril normalized the aldosterone levels in the 6% groups (Table 2). It is possible that the elevated aldosterone levels in programmed rats are a factor in mediating the hypertension with prenatal programming. However, previous studies have shown that maternal low‐protein diet increases expression of NKCC2 and NCC in offspring, it has no effect on renal protein expression of any of the subunits of the epithelial sodium channel (Manning et al. 2002). The fact that 6‐month‐old programmed rats are hypertensive when their aldosterone levels are comparable to controls suggests that aldosterone is not a major factor mediating the hypertension with prenatal programming.

There is indirect evidence that prenatal programming increases the intrarenal renin angiotensin system (Mansuri et al. 2015; Murano et al. 2015). We have recently shown that transient administration of enalapril administered between 21 and 42 days of age and continuous administration of enalapril normalized the elevated urinary angiotensinogen and urinary angiotensin II (indirect markers of the intrarenal renin angiotensin II system (Kobori et al. 2002, 2003; Navar et al. 2003)) found in 6‐month‐old 6% vehicle rats (Mansuri et al. 2015). In the present study the 6% programmed rats did not have an elevated level of renal angiotensin II compared to the 20% rats and while continuous enalapril reduced renal angiotensin II content, a direct assay of the intra renal renin angiotensin II system, transient enalapril did not.

The effect of prenatal insults on histologic markers of glomerular and interstitial injury have previously been performed. In a previous study which studied the effect of a severe prenatal insult, intrauterine food restriction to 50% of control in pregnant rats throughout pregnancy, found greater interstitial fibrosis, and glomerulosclerosis in the restricted group than the control group at 18 months (Lucas et al. 2001). We had previously examined the effect of prenatal programming on glomerular filtration rate at 17 months of age comparing rats whose mothers were administered a 6% protein diet during the last half of pregnancy to those whose mothers were fed a 20% protein diet (Lozano et al. 2015). While we found a comparable reduction in GFR in the programmed rats compared to the control as found in the present study, there were some differences between our studies. We previously found no difference in glomerulosclerosis comparing the 6% group to the 20% group by unpaired Student's *t* test. Performing this comparison between these two groups alone in this study, we found comparable mesangial matrix expansion but greater glomerulosclerosis in the 6% vehicle group compared to the 20% vehicle group. This difference was not statistically significant when all six groups were compared using analysis of variance. In our previous study, as in this one, there was no difference in interstitial fibrosis using picrosirus red staining, and measured using visible as well as polarized light in programmed rats compared to control in both the outer medulla and cortex and no difference in renal collagen content.

This study confirms that prenatal programming causes a reduction in GFR in mature rats (Lucas et al. 2001; Lozano et al. 2015). We had previously shown that cross fostering a neonatal rat whose mother was fed a 6% protein diet to a mother that was fed a 20% protein diet can prevent the reduction in glomerular filtration rate in the 6% group when studied at 17 months of age (Lozano et al. 2015). The present study examined the effect of continuous and transient treatment with enalapril on glomerular filtration rate in 17‐month‐old rats. Enalapril has the effect of decreasing glomerular capillary pressure (Anderson et al. 1985, 1986; Anderson and Brenner 1986), and its salutatory effect on GFR in mature programmed rats is not surprising. The finding that 3 weeks of enalapril therapy administered starting at the time of weaning can prevent the decrease in GFR in programmed rats suggests that the potential deterioration in GFR can be reprogrammed. Gene expression can be affected by epigenetic factors such as histone modification, DNA promotor methylation and microRNAs. Prenatal insults, such as maternal low‐protein diet, have been shown to cause epigenetic changes that affect the renin‐angiotensin system (Bogdarina et al. 2007; Goyal et al. 2010). These epigenetic changes are not set in stone. Losartan has been shown to ameliorate the chronic kidney disease and hypertension seen in db/db diabetic mice and reverse some of the epigenetic changes that may contribute to chronic kidney disease (Reddy et al. 2014). As speculation, it is possible that the brief exposure of enalapril caused epigenetic changes in the programmed rats that prevented the deterioration in renal function. This finding may have implications for small for gestational age and very premature infants if there is a therapeutic window where the risk for hypertension and chronic kidney disease can be attenuated.

## Conflict of Interest

There is no conflict of interest by any of the authors.
